# Bioinformatics Tools for Mass Spectroscopy-Based Metabolomic Data Processing and Analysis

**DOI:** 10.2174/157489312799304431

**Published:** 2012-03

**Authors:** Masahiro Sugimoto, Masato Kawakami, Martin Robert, Tomoyoshi Soga, Masaru Tomita

**Affiliations:** 1 Institute for Advanced Biosciences, Keio University, Tsuruoka, Yamagata 997-0017, Japan; 2 Systems Biology Program, Graduate School of Media and Governance, Keio University, Fujisawa, Kanagawa 252-8520, Japan; 3 Graduate School of Medicine and Faculty of Medicine Kyoto University, Yoshida-Konoe-cho, Sakyo-ku, Kyoto 606-8501, Japan; 4 Department of Environment and Information Studies, Keio University, Fujisawa, Kanagawa 252-8520, Japan

**Keywords:** Bioinformatics, mass spectrometry, metabolome, metabolomics, software development, statistical analysis, systems biology.

## Abstract

Biological systems are increasingly being studied in a holistic manner, using *omics* approaches, to provide quantitative and qualitative descriptions of the diverse collection of cellular components. Among the *omics* approaches, metabolomics, which deals with the quantitative global profiling of small molecules or metabolites, is being used extensively to explore the dynamic response of living systems, such as organelles, cells, tissues, organs and whole organisms, under diverse physiological and pathological conditions. This technology is now used routinely in a number of applications, including basic and clinical research, agriculture, microbiology, food science, nutrition, pharmaceutical research, environmental science and the development of biofuels. Of the multiple analytical platforms available to perform such analyses, nuclear magnetic resonance and mass spectrometry have come to dominate, owing to the high resolution and large datasets that can be generated with these techniques. The large multidimensional datasets that result from such studies must be processed and analyzed to render this data meaningful. Thus, bioinformatics tools are essential for the efficient processing of huge datasets, the characterization of the detected signals, and to align multiple datasets and their features. This paper provides a state-of-the-art overview of the data processing tools available, and reviews a collection of recent reports on the topic. Data conversion, pre-processing, alignment, normalization and statistical analysis are introduced, with their advantages and disadvantages, and comparisons are made to guide the reader.

## 1. INTRODUCTION

Metabolomics or metabolome analysis aims to conduct the simultaneous determination and quantitative analysis of intracellular metabolites. Since metabolomics is concerned with small molecules that are the substrates and products, of cellular activity, it allows to explore in a direct and immediate way the biological system/environment interface. This can be appreciated by the great sensitivity of metabolite levels to subtle pharmacological and toxicological intervention [1-6]. As a consequence, metabolomics is playing an increasingly important role in systems biology, a field that aims to integrate information collected at multiple biological levels. It is now used widely in many applications including microbiology, diagnostic biomarker discovery, toxicological testing, food and beverage analysis, plant and animal phenotyping, and drug discovery and development [7-12].

Nuclear magnetic resonance (NMR) is one of the most commonly used analytical techniques in metabolomics studies [13]. To date, a number of large-scale, studies using NMR have been reported, including blood urine and serum metabolome profiling [14-15]. This technique has been popular in metabolomic studies because of its quantitative nature and high reproducibility. In addition, NMR spectra provide a wealth of biochemical information not available by other means [16-20]. It also has definitive advantage that it can be used in non-destructive ways to enable metabolomic profiling *in vivo* [21-22] and even allow metabolite imaging in biological samples [23-24]. However, the relatively low sensitivity of NMR, and the spectral overlap that often occurs, limits the number and variety of metabolites that can be simultaneously observed. Hyphenated mass spectrometry (MS) methods, such as GC-MS [25], LC-MS [26] and CE-MS [27], currently provide higher sensitivity, and are the leading analytical platform for metabolite profiling [28-31]. Because of the diverse physical and chemical properties (for example, molecular weight, polarity and solubility) of the metabolites contained in typical samples, no single analytical methodology can profile datasets comprehensively. Thus, metabolomics, in the strictest sense, is very challenging, and the term is used broadly to cover approaches concerned with investigating subsets of the metabolome [32]. GC-MS, LC-MS and CE-MS are generally capable of profiling volatile, singly or multiply charged metabolites. Hyphenated MS methods involve the use of a physico-chemical separation method in tandem with a mass spectrometer, which is used for detection. These systems thus produce data that is multidimensional with a time and mass/charge ratio component. The multidimensionality of the data increases the data processing challenges posed by metabolomics. 

Because metabolomics deals with large datasets like other *omics*, sophisticated computational tools are vital for efficient and high-throughput analysis, to eliminate systematic bias and to explore biologically significant findings. In this paper, we review bioinformatics topics in the field of metabolomics, with an emphasis on hyphenated-MS methods, especially LC-MS and CE-MS. As some of these topics have been well reviewed previously [33-51], we emphasize the most recent innovations and developments in the field. In the first part, we review the main data processing steps, including data formats/conversion, feature extraction/detection, comparison of multiple datasets including migration time and mass spectral alignment, signal normalization and identification of metabolites, and quality control (QC). The second part focuses on downstream data analysis of processed datasets, using univariate or multivariate statistical analyses, classification and clustering. We also discuss the standardization of data format, compare some of the leading software tools that implement different algorithms for data processing and discuss data interpretation for different research applications.

## 2. DATA PROCESSING FOR METABOLOMICS ANALYSIS

**Fig. (1):**
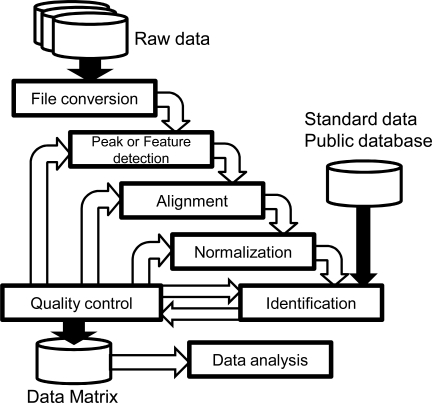
Typical processing flow of MS data in the field of metabolomics. Raw data are sequentially processed in multiple phases, including file conversion, feature detection, alignment and normalization. Standard data and public databases that include metabolite information, such as mass spectrometric data, are used for subsequent feature identification. These processes are then assessed using quality control criteria and the previous phase is repeated if necessary. Once calibrated, the data matrix (aligned detected features across multiple datasets) can be transferred for subsequent data analysis phases.

Typical data processing flow for MS data has been previously reviewed by Katajamaa and Orešič [34], and is now implemented in a variety of software packages [52-57]. The analytical usually flow starts from data conversion, detecting signal peaks, normalization and comparison of multiple datasets to generate a data matrix that includes the detected peaks of all given samples (alignment). The differentiation of signals from noise by interpretation of the mass spectrum and the identification of detected features using, for example, alignments with standard compound data, are also important. Finally, processed data are analyzed using statistical methods and data mining. A recent addition to this straightforward analytical process is the quality control (QC) of data processing. This process does not simply involve the use of QC methods after data processing [58], but rather is used as part of an iterative feedback loop between data processing and QC [59] (Fig. **1**).

The following section introduces recent literature related to 1) data conversion, 2) feature detection, 3) alignment, 4) scaling/normalization, 5) identification, and 6) QC. See also the following references: [58, 60-61].

### 2.1. Data Conversion

Data processing starts with file format conversion from the MS-vendor dependent binary format to more common formats, to allow subsequent processing to be carried out on independent operation systems and software. A common and open framework and data description is important if data are to be shared among laboratories [62-64]. NetCDF and mzXML are the most commonly used file formats to store hyphenated-MS data [65]. Owing to recent rapid improvements in the throughput and resolution of MS, individual data files have become large, which compounds problems associated with the large numbers of datasets handled in metabolomics projects. Although these common file formats simplify data sharing between laboratories, the problem of handling a large number of large datasets remains. While removing small intensity peaks and data compression using irreversible filtering, as can be implemented in mzMine [56] and mzMine2 [52], is the simplest way to diminish data size, they risk distorting subsequent data analysis. Although Mass++ allows the direct import of various binary files provided by MS venders into standard software [66], it merely accesses the binary data through a vendor-provided application programmable interface (API). This dramatically reduces throughput and does not solve the problem of MS-vendor software dependency. Although it cannot be shown directly without access to the source code, most vendor-provided hyphenated-MS instrument binary formats (for example, wiff files and .D formats provided by Applied Biosystems and Agilent Technologies Inc.) can be estimated to contain a series of mass spectra data, since mass spectra are usually collected in this way. This data structure results in much longer data access times to output a chromatograph or an electropherogram if the data points included in the mass spectra are not unique over the chromatograph or electropherogram. To solve these size and structure problems, we developed a compact binary file format that facilitates rapid access to chromatographs or electropherograms and mass spectra [67]. Although there is currently a trade-off between facilitating quick data access and the availability of a generic file format, the development and standardization of file formats that fulfill the requirement for rapid access should be a priority.

### 2.2. Feature Detection

In the typical analytical flow, three-dimensional data incorporating retention or migration times, *m/z* and intensity data are first converted to piles of two-dimensional chromatography/electropherograms, by integrating data points within a specific range along the *m/z* axis (ion extraction or data binning). Second, background reduction (or baseline removal) and smoothing of the data are conducted to reduce false positive detection. Third, local maxima are found as peak top candidates, or a mathematical model is fitted to find peak-like shapes within the chromatographs or electropherograms. These are used to identify peaks over a user-specified threshold, which may be in the form of a peak height, peak area or signal-to-noise (S/N) ratio [52-54, 67-68]. Although wavelet transformation and Gaussian-curve fitting (or matched filter) is a commonly used means to distinguish signal from noise [53, 68], fully automatic processing remains difficult owing to the complex peak shapes often observed in LC-MS and, in particular, CE-MS. Interactive tuning of the algorithms is therefore often required [52, 67]. Other options are to identify peaks at matched locations (*m/z* and time), even under the initially-defined threshold after the alignment process [69]. Such feedback procedures and QC will be discussed further in section 2.7.

### 2.3. Alignment of Multiple Data Sets

The alignment of multiple datasets, i.e. the elimination of retention or migration times shifts between datasets, is a central topic of data processing in the metabolomics field, and is associated with specific technical difficulties. Therefore, many alignment techniques have been developed [70]. The retention time variance of GC-MS and LC-MS datasets is non-linear [71], and thus multiple sophisticated time correction methods have been developed. The alignment of CE-MS data is especially difficult because of the low reproducibility of migration times [54], and robust and versatile alignment procedures are therefore required. Here, we review the three major alignment algorithms used for the temporal dimension. In addition, the normalization of mass/charge ratio (*m/z*) calculated by MS is also introduced.

#### Time Correlation Optimized Warping

Time correlation optimized warping (COW) divides chromatograms into small segments and shifts individual segments to maximize the correlation coefficient between a reference and test chromatograph. The algorithm itself has inherent problems; a larger number of segments leads to greater accuracy, but raises the risk of dividing the targeted metabolite peaks. To optimize the degree of segmentation, the use of heuristic and global optimization processes, such as genetic algorithms, has been proposed [72]. To date, benchmark tests with only small numbers of peaks have been performed [72], and the method should be evaluated using data with a large number of peaks, observed by high resolution MS.

#### Parametric Time Warping 

The parametric time warping method aligns a given chromatogram with a reference chromatogram using second degree polynomial functions, called warping functions [73]. Coefficients in warping functions are optimized to minimize the time difference between selected matched peaks in reference and aligned chromatograms. Thus, the method relies on the presence of a number of known matched peaks among the samples to be aligned. Although the addition of internal standards (IS) is the most simple way to achieve this, it has several disadvantages: (i) suitable IS compounds must be carefully selected, for example the IS compounds must not normally be present in the samples; (ii) additional sample preparation is required; and most significantly (iii) the added IS may cause ion suppression effects and degrade the quantitative reliability of the observed profiles. Despite these problems, rapid computation time is an important advantage of this method. Lower flexibility and accuracy has been reported for this method in comparison with COW and dynamic time warping (DTW) [70]. 

#### Dynamic Time Warping

DTW finds the matched peaks among multiple datasets automatically to produce warping functions. Dynamic programming (DP) has historically been used in homology searching of genes or genomes, and has been used for matching peaks [74]. The parameters that characterize DP results, such as gap penalty, make this method parametric. Thus, empirical reiterative multi-step optimization of these parameters has been used in CE-MS data processing software [54] and interactive graphical user interfacing [67]. In contrast, recent modifications to DTW using multiple chromatograms with different *m/z*, instead of one-dimensional information available from total ion chromatography, reduced the impact of the parametric problems embedded in the original DTW algorithm [75].

#### Calibration of Mass Values (m/z Alignment)

Exact masses (mass-to-charge ratio (*m/z*) values), produced by detectors in time-of-flight (TOF)-MS instruments are usually calculated based on online calibration with one or more reference substances that are co-injected with the sample. This is known as the mass lock system [76]. The *m/z* values detected for individual peaks fluctuate depending on several factors, including temperature, the abundance of ions simultaneously entering the MS, and the processing ability, type and specifications of the MS detector [77]. Thus, the data acquired should be further calibrated. Typically a calibration curve generated using the peaks of known *m/z* is applied to correct *m/z* values of other peaks of interest (offline or software calibration) [78-80]. The *m/z* values are intricately calibrated for the whole chromatograph or electropherogram time axis, since the factors influencing *m/z* shifts can change even during the course of a single run [81]. In addition, *m/z* value correction can be carried out using peak intensities relative to the intensities of internal standards [82], using the location of background noise observed throughout the measurement [83], and using statistical approaches with multiple datasets [69]. Ideally, these methods should be integrated to optimize *m/z* normalization.

### 2.4. Scaling and Normalization

The elimination of unwanted systematic bias, while maintaining genuine biological differences in the observed datasets, is essential for subsequent analyses to identify significant metabolites. The systematic bias derived from variation in sample concentration, especially when handling biofluids such as urine, blood and saliva samples, must be removed. Deviation in signal intensities due to measurement errors, for example poor MS sensitivity, must also be removed. To address the former problem, metabolomic analyses typically use endogenous metabolites, for example creatinine, to normalize overall urine metabolite concentrations [84]. However, this method is not always sufficient to eliminate systematic bias, and a recent mouse metabolomic study revealed a correlation between overall urinary metabolites and several physical parameters, such as age and weight [85]. The latter bias is generally removed using two approaches. Despite the increased technical complexity of sample preparation, the use of internal standard compounds added to the sample before or after extraction is the most common approach. The use of multiple internal standards to normalize closely eluting peaks with similar *m/z* values has also been reported [86]. Otherwise, normalization methods based on several statistical models (unit norm [87] median [88] and quantile [58]), scaling methods (auto scaling, range scaling, Pareto scaling, vast scaling and level scaling) [61], and data transformation (log and power) have been widely used. These methods are, however, inferior to the internal standard-based methods [58].

### 2.5. Identification of Metabolites

Global metabolic profiles or fingerprints that do not necessarily assign observed features to particular metabolites can be very powerful means of classifying and directly comparing samples. They highlight metabolomics as providing a global molecular signature allowing us to discriminate groups of samples in contrast to more conventional comparisons based on single metabolite. However, metabolite identification from spectral data remains indispensable for providing mechanistic insight into specific cellular or disease processes and in quality control/assurance industry, for example. The accurate identification of a compound usually requires the ability to match candidate spectra with standard compounds run under the same conditions. Matching to either externally or internally applied standards has been commonly used, the latter making use of isotopically labeled standards or samples. However, the lack of readily available standard compounds remains a major obstacle to confirming the identity of observed compounds. The purification of compounds from complex samples allows access to standards; however, this can be an expensive and time-consuming process. Several tools that estimate compound composition using isotope distribution or fragmentation patterns in the mass spectrum have been developed [89-92]. Databases that include a large number candidate compounds are also indispensable (see review [43]). A theoretical study estimated that the mass spectral information available from mass spectrometers with accuracy approaching 1 ppm, such as TOFMS, is not sufficient to identify peaks without a matched standard compound, as multiple candidate compounds are often retrieved from the large public databases [93]. The Human Metabolome Project has already identified more than 4,000 putative endogenous metabolites from human serum using GC-MS, LC-MS and NMR profiles with computer-aided literature mining [12]. Many studies thus use tandem MS, which generates more informative spectra including many fragment peaks, for compound identification [94-95]. Efforts have also been made to use retention time information to reduce the number of possible candidates. These efforts are based on reverse engineering techniques [96-99] or theoretical simulation [100], which predict the retention/migration times from the metabolite structure. The quantification of observed peaks in the absence of matched standard compounds is also difficult, but computational prediction techniques have been developed [101]. The combined use of such computational methods can greatly reduce the number of candidates and aid metabolite identification.

### 2.6. Quality Control of Data Processing

A number of algorithms have been developed for data processing, especially for peak detection and alignment, and various parameters can be used to characterize the quality of data processing [59]. The selection of the best algorithm, and the best parameters, to analyze the datasets obtained is not an easy task. Thus, QC evaluation based on various benchmark tests is important to understand the features of each algorithm and their parameters [102].

A comparison of peak detection algorithms of LC-MS data using centWave [68], matched filter implemented in XCMS [53] and MZmine [56] showed that there was only a partial overlap in the results obtained with these methods, and a number of peaks were only detected by one software (not overlapped) [68]. Even with the same algorithm, the use of different parameters strongly affected peak detection performance [58]. Evaluation of the alignment of LC-MS data using six freely available software packages, including XCMS [53], MZmine [56], msInspect [103] and OpenMS [55], concluded that no single software perfectly aligned the datasets [104]. The annotation of metabolite identities using fixed confidence thresholds has been recommended for data reporting, as has quantitative assessment of the annotation quality using the false discovery rate (FDR) [105]. Another approach is to provide a sophisticated graphical interface that enables specific steps of data processing to be rerun using different parameters [52]. Scripting tools may also be used to accelerate the optimization process and to minimize the need for user interactivity. Another possible means to improve performance entails the development of an iterative analytical framework with machine learning methods that allow the program to be trained to tune parameters using the difference between automated and manual data processing [59]. It is evident that subsequent statistical analysis will benefit if care is taken at the processing stage, and that automatic data processing for peak detection, alignment and annotation remain far from perfect.

## 3. DATA ANALYSIS IN METABOLOMICS

**Fig. (2):**
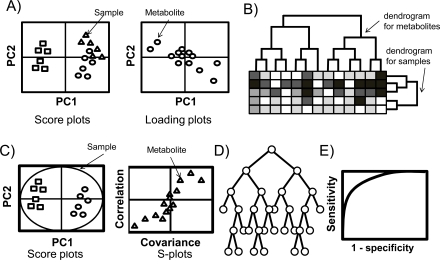
Typical data analysis methods used in the field of metabolomics. Score plots in PCA **A**), dendrograms of clustering **B**), score plots and S-plots of PLS-DA **C**), random forests model **D**), and ROC curve **E**).

Once a data matrix has been produced from raw data, subsequent steps usually involve different forms of statistical analysis and data mining to allow the identification of samples or variables (metabolites) that capture the bulk of variation between datasets and that may represent candidates for biologically meaningful variables. Typical analyses of metabolomic data consist of two phases; initially an overview of the given datasets is generated using multivariate analysis and individual peaks are subsequently graded by univariable analysis. Here we briefly introduce several univariable and multivariate analyses, and classification and assessment methods that are widely used in analyzing MS-based metabolomics datasets (Fig. **2**). Selected recent applications are then introduced. See also the recent reviews [37, 43].

### 3.1. Principal Component Analysis

Principal component analysis (PCA) is an unsupervised statistical analysis that is probably the most widely used statistical tool in metabolomics studies. PCA converts high-dimensional data into fewer dimensions, by projecting the data into a reduced dimensional subspace, while maintaining as much variance from the original data as possible [106-108]. The procedure is repeated until the datasets can be presented usually within two or three dimensions. This facilitates visual inspection of the distributed samples in principal component (PC) space, using score plots [33]. The Euclidian distance between individual samples in score plots reflects the degree of systematic variation in metabolite profiles among samples, and loading plots show the contribution of individual metabolites to each PC (Fig. **2A**). Prior to the development of more effective data analyses, such as clustering, pattern recognition or classifications, the vast majority of metabolomic studies used PCA as a first exploratory step [37].

### 3.2. Cluster Analysis

Clustering analysis is a statistical method that involves dividing observed datasets into several subclasses or clusters based on a selected statistical distance function. There are two types of clustering algorithms: hierarchical and non-hierarchical methods. Both algorithms partition the observed datasets into subgroups so that datasets with similar metabolomic profiles are placed in each subgroup [33]. Hierarchical clustering (HCL) (Fig. **2A**) aligns datasets by generating dendrograms using the following procedure: 1) calculate the similarity of the two samples using a specific metric, such as Pearson correlation, Euclidean, mutual information and covariance values; 2) align the most similar samples as neighbors or pair them as a single cluster; and 3) reiterate step 1 and 2 until all samples are aligned [33]. Non-hierarchical clustering (non-HCL) also divides data into clusters but without any hierarchical organization. The K-means and fuzzy c-means methods are typical examples of non-HCL [33]. In the K-means method, k data points are initially randomly chosen to be close to the mean of each cluster, a new mean is then calculated for each cluster and the patterns are reassigned to the new means. This process is repeated until the cluster means are such that no pattern moves from one cluster to another [109]. The K-means method assigns each datapoint into only one cluster while the fuzzy c-means method allows data to be assigned to multiple clusters [110]. Fuzzy c-means also calculates the probability of a datapoint belonging to each cluster [111]. These analyses are widely used when the number of clusters for the samples is unknown, and can be used for one-time snapshot profiling as well as time-course data.

### 3.3. Partial Least Squares Analysis

Partial least squares (PLS) (Fig. **2C**), a regression-based method, builds a low-dimensional sub-space based on linear combinations of the original X variables. It makes use of additional Y information by adjusting the model to capture the (Y)-related variation into the original X variables [37]. PLS is particularly useful when fewer observations (samples) are available than measured variables (metabolites). In metabolomics, PLS-based classification and PLS-discriminant analysis (PLS-DA) have been widely used to sharpen the separation between groups or observations. This is achieved by rotating PCs to maximize the separation between known classes, and to elucidate the variables that carry the class separating information [33,112-113]. Similarly to loading plots in PCA, S-plots visualize both the covariance and the correlation between metabolites and the modeled class designation. The S-plot therefore helps to identify statistically significant and potentially biochemically significant metabolites, based both on contributions to the model and their reliability [114]. Despite its powerful ability to separate classes, care must be taken during fitting of PLS-DA to the training detaining datasets, which exaggerate generalization ability. Usually cross-validation or permutation tests are used to assess the ability of the trained PLS-DA model [115]. Orthogonal projections to latent structures (OPLS)-DA, an extension of PLS-DA featuring an integrated orthogonal signal correction filter to remove variability not relevant to class separation, has been used increasingly owing to its robustness against noise [21,116].

### 3.4. Random Forests

Random forests (RF) is a relatively new machine learning method typically used to discriminate two groups (Fig. **2D**). The fundamental concept of RF is to allow data structures to be understood without dimensional reduction, and this method is therefore different from conventional methods such as PCA and PLS-DA. This classification algorithm was developed by Leo Breiman [117] and uses an ensemble of classification trees. Each of the classification trees is built using a bootstrap sample of the data, and at each split, the candidate set of variables is a random subset of the variables. Thus, RF uses both bagging (bootstrap aggregation), a successful approach for combining unstable learners, and random variable selection for tree building. Each tree is unpruned (grown fully) so as to obtain low-bias trees. At the same time, bagging and random variable selection result in low correlation of the individual trees [118]. The algorithm yields an ensemble that can achieve both low bias and low variance (by averaging over a large ensemble of low-bias, high-variance but low-correlation trees) [119].

### 3.5. Conventional Statistical Analysis

Because metabolomics generates data on multiple (dozens or hundreds) different metabolites, global overview methods that take into account the possible correlations between variables are the main tools used. However, when used appropriately, monovariate methods can also provide useful insight and remain widely used, especially for secondary biomarker analyses.

Although multivariate classification methods are often used to identify biomarkers, the discrimination of individual metabolites is usually assessed by conventional univariate statistical tests, such as Student’s t-test and the Mann-Whitney test for two classes, or ANOVA and Kruskal-Wallis for multiple classes (≥3). Dependency or correlations between metabolites, inadequate sample size, and large FDR due to multiple hypothesis testing must be taken into account when applying these methods [120]. Corrections of the *p*-value and/or calculation of false discovery rates must be carried out to limit the number of false positives that increase linearly with the number of variables [120]. Multivariate analysis has the advantage of considering the general patterns in the whole dataset, but it introduces additional challenges and sources of variability owing to the necessary data pre-treatment and scaling used to analyze all variables at once [61]. Thus, biomarkers should be rigorously evaluated by a combination of these statistical analyses and several validation methods, such as cross-validation and bootstrap analysis [121]. Recently, the FDR and receiver operating characteristics (ROC) methods have been frequently used to identify significantly different metabolites in the given classes.

The FDR method [122], is commonly used in gene expression analyses, and is now also used in metabolomic studies, [11], where a large number of variables are analyzed simultaneously, and thus multiple comparisons are conducted. In practice, FDR establishes a threshold for the significance level (*q*-value) that can be expected to represent false positives among all significant hypotheses to reject optimistic significance. To account for multiple comparisons, each FDR is estimated by the product of the significance level (Type I error rate) and the number of null hypotheses tested, divided by the number of null hypotheses rejected [123].

A ROC [124] curve is a statistical representation that simultaneously expresses both sensitivity and specificity to separate binary class datasets, for example to discriminate healthy control and patient datasets. The curve is plotted by fractions of sensitivity as the Y-axis vs. fractions of false positive rate (1- specificity) as the X-axis (Fig. (**2E**)). The test is used to differentiate performance of one or a combination of biomarkers; an area under the curve (AUC) of 1.0 indicates perfect separation without any false negatives or false positives, while an AUC of 0.5 is equivalent to random separation only. 

AUC evaluates only the rank of the metabolites associated with the given classes, and therefore it does not count fold-change or the concentration itself. Meanwhile, FDR evaluates the relative significance of the metabolites in a large group of metabolites. Thus, the use of a combination of different methods, along with multivariate analyses, can achieve more efficient screening than any single method.

### 3.6. Data Mining Analysis

In addition to classification methods, other data mining methods have also been used in metabolomic data analyses to discriminate two classes, for example support vector machine (SVM) [125-126], artificial neural networks (ANN) [127] and decision tree [128]. ANN has been particularly widely used for various applications in MS-based studies, including in metabolite identification [97], classification [129], optimization of separation parameters [130] and QC of data processing [59] (see review [131]). In comparative study, a class of LC/MS peaks was predicted by four data mining techniques, k-NN, SVM, PLS-DA and Naïve Bayes, and revealed that the former two methods performed better than the latter two [132]. However, it is usually difficult to select the best method for the analysis of a given metabolomic dataset *a priori*, and the development of a pipeline with multiple analytical tools is therefore necessary. Visualization of metabolomic data in a pathway form also requires several data mining techniques. Small relevance and conditioned metabolic pathways have been predicted and then merged to generate pruned networks [133]. Small sub-pathways were estimated with only relevant nodes, for example metabolite and enzymes, to reduce complexity and to enhance interpretability [134]. Both of these method attempts to find new relevant connections, rather than to assign the observed data to known maps.

## 4. VISUALIZATION AND DATA SHARING

Here we discuss data visualization to facilitate the interpretation of large metabolomic profiles. Data standardization is also discussed to realize open and shared access to metabolomics technologies.

### 4.1. Visualization of Metabolomics Data

Data visualization using a heatmap or a pathway form facilitates comprehension of the metabolomic change/response to the experimental setting. MetaboAnalyst visualizes experimental metabolomic data using heatmap visualization and offers common statistical analyses, such as PCA, PLS-DA, and HCL [135-136]. Pathway Project [137] visualizes data in the form of several graph types, such as bar graphs, time-courses and simple circles corresponding to metabolite concentration at the metabolite node on the KEGG pathway [138]. Similar web-based network visualization tools for BioCyc [139] are also available [140]. Both tools take advantage of Google Map API zoom and search functions, which can be helpful when looking for interesting details in large metabolomic datasets. The editable pathway tool is also useful when new molecular interactions that are not available in public database are to be explored [141].

### 4.2. Standardization of Metabolomics Data Reporting

In addition to the standardization of raw file format and data processing tools, the standardization of the reporting of metabolomic data information has also received attention. This would facilitate experimental replication, interrogation and comparison over multiple investigators and laboratories. The Metabolomics Society has formed five working groups, biological context metadata, chemical analysis, data processing, ontology and data exchange, to establish guidelines for reporting standards [142]. The Chemical Analysis Working Group, part of the Metabolomics Standards Initiative, proposed a set of minimum information that should be provided when reporting chemical analyses, and these included metadata from MS and NMR data, sample processing protocol, data processing, metabolite identification, and even unknown metabolites in the obtained dataset [143]. Attempts to define standards for data reporting have been made but unfortunately are still not widely used [35, 142-143]. To maximize the value of metabolomic datasets, it is important that data is made publicly available in formats, and with metadata, that are widely accepted as standard. In this sense, the field of metabolomics lags behind genomics and proteomics. Some of the reasons for this slow adoption of standards include the heterogeneity of analytical platforms and vendors, and the complexity of sample processing, which remains the focus of ongoing investigation. A Metabolomics Standard Initiative was recently initiated by the Metabolomics Society, and aims to develop standards for data exchange, ontology and guidelines for data reporting to solve some of the current issues (http://msi-workgroups.sourceforge.net/).

## 5. SOFTWARE TOOLS

A number of free software packages are already available for the processing and analysis of metabolomic data, and Table **1** gives a sample directory of these. Both web services and desktop applications are available. The table is not necessarily exhaustive, but should help to identify commonly used solutions. Several statistical tools listed were designed for NMR data analyses but might be also useful for MS data analyses. Here, we focused only on tools used specifically in metabolomics studies, and did not review free or commercial generic software for multivariate analysis or other standard statistical analysis. We emphasize mainly tools for pre-processing and data visualization. Moreover, details of these packages are not reviewed here, and the reader is referred to the original publication or project web site for more information.

## 6. APPLICATIONS

Here, the use of statistical methods in several applications is discussed. Note that several of the statistical analysis applications introduced here used NMR data. The same multivariable techniques can technically be used for MS data analyses, but it should be noted that MS-data includes a larger number of variables (metabolites) and therefore more redundant variables. However, appropriate statistical analyses and MS data may provide more powerful insight into biological context.

PCA and PLS-DA have been the most popular and widely used analyses in metabolomic studies. Although PCA can visualize the similarities and differences in the observed data with unknown classes, it is generally used as a weaker classification tool for class known problems. It is therefore generally used as a first screening method for classification problems, prior to PLS-DA. For example, while PCA was able to give adequate separation resolution of various conditions, for example smokers and non-smokers in a salivary metabolite profile, PLS-DA was subsequently used to maximize resolution [144]. A similar approach was adopted for the discrimination of lung cancer sufferers using urine metabolomic profiles [116] and pancreatic cancer using serum metabolomic profiles [145]. HCL has also been used to assess data structure by aligning datasets based on their profile’s similarities [146-148], and this method is often used to classify samples with known classes, similarly to PCA. It has been applied to biomarker discovery, to classify control and patient groups, with key branches in its dendrogram indicating biomarker candidates [149]. Although this example was not a metabolomics application, a particularly successful example of HCL involved the clustering of gene expression in breast cancer, which suggested the existence of a new subtype of breast cancer in addition to the known classes [150]. The assessment of the analytical results of these methods can only be performed with known classes, and new findings should be analyzed further once consistency between results and known classes has been confirmed.

The over-fitting of a developed model to a given dataset should be carefully avoided, especially when using MS data, since it usually involves a large number of variables and small sample numbers. RF is expected to be a useful classification method when we use such datasets. Because the algorithm itself does not limit the application, RF has been used for biomarker discovery in urine metabolomic profiles from breast cancer patients [125] and in plant applications to explore genotype-dependent variables in metabolomic profiles in *Arabidopsis* and potato [151-152]. When RF and margin-based classifiers, such as SVM and PLS-DA, were compared, RF and SVM were found to have similar accuracy and both were slightly better than PLS-DA [125]. However, the accuracy of the model trained on the given dataset is not the only important factor. Validation, which involves confirming the generalizability of the model’s accuracy and the significance of selected variables in similar experiments, is important when such discriminate models are used. SVM and PLS-DA can also be used to rank the significance of variables constitutive to the models, while RF does not explicitly maximize the margin, which makes the trained model unbiased to the given datasets and is directly related to the generalizability [151]. Although several techniques to evaluate generalizability are known, including the permutation test, bootstrap test and cross-validation [115], rigorous assessment has indicated that normal cross-validation is insufficient and overfitting may remain a problem [153]. Thus, careful and multilateral evaluation of the developed model is necessary.

After multivariate analysis, individual metabolites or sets of metabolites are usually accessed using univariate analyses. As ROC is a conventional statistical method that has been widely used for medical diagnosis problems, it has become popular in biomarker discovery applications. Multiple logistical regression models, composed of multiple metabolite markers to discriminate liver diseases [154] and oral cancers [155], were assessed using AUC values calculated from ROC. This revealed the discrimination possible when only a few metabolite sets are used, rather than all available data, which is used in PCA and PLS-DA. Approaches using all available metabolites are appropriate when studying overall variation, but are not useful for clinical usage, for example in the development of diagnosis techniques using a single or a few markers. Thus, integrative analyses using multivariate analysis, feature selection, and assessment of individual or a few markers are standard techniques that are useful for general purposes.

As should be apparent, multiple solutions exist for data processing, some of which are capable of performing most or all steps from raw data to statistical analysis, while others are specialized for certain steps or visualization. The selection of a data analysis solution is not straightforward and will depend on the analytical platform, the experimental design and data type, and on computational infrastructure, among other things. This review gives an overview of the options that can be chosen from, and highlights recent efforts to integrate these solutions to generate simple, yet powerful methods for the user. The field of data analysis for metabolomics is still rapidly evolving, and ongoing efforts are likely to produce further progress. There is a need for greater interchangeability and interoperability between tools, and unfortunately the profusion of new and interesting tools originating from numerous small groups often tends to limit this goal. Developers should consider these factors when promoting particular solutions. This will stimulate data sharing and exchange, and therefore improve adoption by a community of users who are often overwhelmed by a range of possibilities, and who may therefore tend to stick to tools that emphasize usability rather than quality or performance.

In this review article, we reviewed multiple tools for processing and analysis of MS data. Multiple metabolomics platforms together with the appropriate data processing and analysis tools can allow us to identify discriminating features in a set of samples, with multiple applications in research, diagnosis, etc. However, beyond class discrimination, understanding the biological mechanisms responsible for the variance in observed profiles remains an important issue. For this, the constant development and improvement of computational techniques for metabolite identification, accurate quantification, data integration, and pathway visualization is important and will continue to be the focus of bioinformatics efforts in the coming years. 

## CONCLUSION

Remarkable improvements in analytical instruments, including MS, have enabled the profiling of metabolites with increasingly high throughput and high precision. Bioinformatics, which facilitates the interpretation of the output of these instruments, is essential to the successful analysis of large dataset metabolomic applications. Tool development must keep up with the improvements in analytical instruments and thus represents an important challenge, but has great potential to add value to metabolomic datasets.

## Figures and Tables

**Table 1.** Software List for Metabolomic Analysis

Name	Main Application	Specific Features	Ref.	License	User Interface
OpenMS	Raw data processing	C++ libraries for MS data processing, including feature detection and protein/peptide identification	[57]	Lesser GNU Public License (LGPL)	C++ library
CDK-Taverna	Workflow	A workflow based data processing library for cheminformatics	[156]	LGPL	Plug-in of Java
Metabonomic Package	Statistical analysis of NMR data	Multivariate analysis, such as PCA, PLS, k-nearest neighbor classification, neural networks.	[127]	GPL	R language*))
metaXCMS	Importing XCMS output	Post processing of XCMS for comparison of multiple (≥3) classes and visualizing statistical analyses.	[157]	Free	R language*) and GTK
XCMS	Processing LC-MS raw data	R module for data processing, including feature detection and peak alignment	[53]	Free	R language*)
XCMS2	Importing tandem mass spectrometry (MS/MS) raw data	Processing of tandem mass spectrometry data for metabolite identification and structural characterization	[158]	Free	Plug-in of R language*)
MeDDL	Data processing of LC-MS and GC/MS data	A Matlab script for data processing and visualizing multiple datasets.	[159]	Free	Matlab script
MetaScape	Pathway visualization / statistical analysis	A Cytoscape plug-in for visualizing and interpreting metabolomic data in the context of human metabolic networks	[160]	Free	Plug-in of Cytoscape
MetaboliteDetector	Importing NetCDF and FastFlight GC-MS data	Comprehensive analysis, including chromatogram compression, feature detection, alignment and compound identification.	[161]	GNU public license (GPL)	Local application (GUI)
MetAlign	Importing many common formats, including Masslynx, Xcalibur, netCDF, and the old-style HP/Agilent format of GC-MS / LC-MS data	Interface-driven data processing program. Includes baseline correction, smoothing, feature detection and alignment	[162]	Free	Local application (GUI)
MAVEN	Data processing of LC-MS and pathway visualization	Tools for all aspects of data analysis, from feature extraction to pathway-based graphical data display	[59]	Free	Local application (GUI)
LIMSA	Data processing / mass spectrometric lipidome data	Tool finds and integrates peaks in a mass spectrum and matches the peaks with a user-supplied list of expected lipids.	[163]	Free	Local application (GUI)
centWave	Data processing of LC-MS data	Detection of close and partially overlapping features; also has the highest overall recall	[68]	Free	Local application (GUI)
mzMine2	Data processing of MS data	Modular framework for processing, visualizing and analyzing mass spectrometry-based molecular profile data	[52]	Free	Local application (GUI)
JDAMP	Data processing of CE-MS data	Data processing, alignment, differential display	[67]	Free for academic users	Local application (GUI)
CytoScape	Pathway visualization / statistical analysis	Software for the visualization and analysis of biological networks	[164]	Free	Local application (GUI)
metaP-server	Statistical analysis, database searching, pathway visualization	A web-based metabolomics data analysis tool	[165]	Free	Web
MetDAT	Statistical analysis, database searching, pathway visualization	A modular and workflow-based free online pipeline for mass spectrometry data processing, analysis and interpretation	[166]	Free	Web
ChromaA	Alignment, chromatography-mass spectrometry	Signal-based retention time alignment for chromatography-mass spectrometry data	[167]	Free	Web
MZedDB	Data processing	Interactive m/z annotation tool	[92]	Free	Web
Pathway projector	Pathway visualization	A Web-based zoomable pathway browser that uses KEGG atlas and Google Maps API	[137]	Free	Web
MetPA	Pathway visualization / statistical analysis	A web-based metabolomics tool for pathway analysis and visualization	[168]	Free	Web
MetExplore	Pathway visualization	A web server to link metabolomic experiments and genome-scale metabolic networks	[169]	Free	Web
MSEA	Pathway visualization	A web-based tool to identify biologically meaningful patterns in quantitative metabolomic data	[170]	Free	Web
MetabolomeExpress	Pipeline for data processing and statistical analysis of GC/MS data	Data processing, statistical analysis (e.g. HCL), metabolite identification and heat map visualization	[171]	Free access for non-commercial and academic users	Web
Chromaligner	Alignment of LC-MS data	Alignment of LC-MS chromatographs using the COW algorithm	[172]	Free access	Web

*) R language (http://www.r-project.org/).
